# Clear Cell Renal Cell Carcinoma Recurrence Three Decades After Nephrectomy Presenting With New-Onset Diabetes

**DOI:** 10.7759/cureus.104020

**Published:** 2026-02-21

**Authors:** Alexander Ponce, Victoria Comfort, Samuel Peeples

**Affiliations:** 1 Internal Medicine, William Carey University College of Osteopathic Medicine, Hattiesburg, USA; 2 Internal Medicine, Baptist Hospital, Jackson, USA

**Keywords:** hemoglobin a1c, metastases, renal cancer, renal clear cell carcinoma, type 2 diabetes, weight loss

## Abstract

Clear cell renal cell carcinoma (ccRCC) is a common cancer that comprises the majority of all renal cancers. Patients typically present with hematuria, flank pain, and constitutional symptoms like fever, fatigue, and weight loss. ccRCC is often managed with chemotherapy, and, under some circumstances, the removal of a kidney. Although recurrence is possible following removal of the kidney, it is uncommon for a recurrence to occur after several decades. An 80-year-old man with a history of hypertension, hyperlipidemia, and ccRCC status post nephrectomy 33 years prior presented with two months of unintentional weight loss, fatigue, decreased appetite, and urinary frequency. Serial weights confirmed a 24-pound (12%) weight loss. Physical examination was notable only for mild epigastric tenderness. Laboratory evaluation demonstrated new-onset severe hyperglycemia with a hemoglobin A1C of 13.7% and serum glucose of 351 mg/dL, without prior documented diabetes. Given the rapid onset of hyperglycemia and significant weight loss, computed tomography imaging revealed a 9.2 × 6.4 cm pancreatic mass with additional lesions in the right lung and liver, initially concerning for metastatic pancreatic malignancy. Subsequent positron emission tomography and biopsy of the pancreatic head confirmed metastatic ccRCC. The patient was initiated on systemic therapy with axitinib and pembrolizumab, resulting in interval reduction of the pancreatic mass and resolution of pulmonary metastasis on follow-up imaging. Surgical excision of the pancreatic lesion has been scheduled in light of treatment response. This unique presentation and unlikely recurrence illustrate a rare case of ccRCC. Moreso, while diabetes is a known risk factor for ccRCC, the association between ccRCC and new-onset diabetes has rarely been documented. This case seeks to reinforce the importance of considering metastatic disease in patients with unexplained metabolic abnormalities and a remote history of renal cell carcinoma.

## Introduction

Clear cell renal cell carcinoma (ccRCC) is a malignancy arising from the renal parenchyma and represents the most common histologic subtype of renal cell carcinoma (RCC). Kidney cancer accounts for approximately 3-4% of adult malignancies, and ccRCC comprises roughly 70-80% of renal cancers [1]. When symptomatic, ccRCC commonly presents with hematuria, flank pain, constitutional symptoms (fever, fatigue, weight loss), and, less commonly, a palpable flank mass reflecting tumor-associated renal enlargement [2].

The most frequent sites of metastatic spread include the lungs, bone, liver, and regional lymph nodes, with dissemination occurring through hematogenous and lymphatic pathways, including retroperitoneal lymphatic involvement [1,3]. Pancreatic metastases by ccRCC can occur and account for 2.8% of metastases [4]. Following definitive management with nephrectomy (with systemic therapy as indicated), recurrence risk is variable and depends largely on stage and pathologic risk factors; reported recurrence rates within the first five years range from approximately 20 to 40% in localized disease cohorts [3]. Although most recurrences occur within the first five years, rare cases of late relapse occurring decades after nephrectomy have been reported [5].

Diabetes mellitus has been associated with an increased risk of ccRCC in epidemiologic studies, and metabolic conditions such as diabetes and obesity have been proposed as contributory risk factors [6-8]. Additional established risk factors include cigarette smoking and hypertension [6-8]. Few reports have described rare cases of ccRCC occurring in the setting of long-term hemodialysis with metabolic abnormalities including glucose dysregulation, described in select contexts [6,8]. This report discusses the clinical significance, diagnostic challenges, and potential metabolic manifestations associated with late recurrence of ccRCC. This case highlights a rare, extremely delayed recurrence of ccRCC presenting as new-onset diabetes temporally associated with pancreatic metastasis.

## Case presentation

An 80-year-old man with a past medical history of hypertension and hyperlipidemia presented with unintentional weight loss and fatigue over the past two months. The patient reported a history of ccRCC diagnosed 33 years prior, for which he underwent nephrectomy, with no evidence of recurrence since that time. His appetite had decreased, and he had urinary frequency. The patient noticed a gradual decline in energy levels, describing a general sense of weakness that began to affect his usual daily activities. Despite these symptoms, he denied fever, chills, night sweats, or any recent infections, and he had not made intentional changes to his diet or medications.

Serial weight measures confirmed a 24-pound weight loss over two months. Physical examination revealed a chronically ill appearing male with a normal cardiopulmonary exam. The abdominal exam revealed no palpable masses, but mild epigastric tenderness to light and deep palpation was noted. 

Laboratory evaluation (Table 1) included hemoglobin A1C, comprehensive metabolic panel, and complete blood count. Results showed a hemoglobin A1C of 13.7%, with no prior A1C recorded. The comprehensive metabolic panel showed a potassium of 5.2 mmol/L, glucose of 351 mg/dL, calcium of 10.5 mg/dL, and alkaline phosphatase of 153 U/L. Complete blood count was normal. Urinalysis showed no hematuria or pyuria but had 3+ glucose and 1+ protein.

**Table 1:** Lab results Reference ranges may vary by institution. Only laboratory values available in the chart were retrievable and are presented.

Panel	Test	Result	Reference Range
Complete Blood Count Results	White Blood Cell Count	7.5 K/uL	4.0 - 11.2K/uL
	Red Blood Cell Count	5.06 M/uL	4.40 - 6.00 M/uL
	Hemoglobin	14.8 g/dL	13.5 - 18.0 g/dL
	Hematocrit	43.1%	40.0 - 52.2 %
	Mean Corpuscular Volume	85.2 fL	80 - 100 fL
	Mean Corpuscular Hemoglobin	29.2 pg	27.0 - 33.0 pg
	Mean Corpuscular Hemoglobin Concentration	34.3 pg	31.0 - 36.0 pg
Comprehensive Metabolic Panel Results	Sodium	137 mmol/L	134 - 144 mmol/L
	Potassium	5.2 mmol/L	3.5 - 5.2 mmol/L
	Chloride	101 mmol/L	96 - 106 mmol/L
	Carbon Dioxide	32 mmol/L	20 - 29 mmol/L
	Glucose	351 mg/dL	70 - 100 mg/dL
	Blood Urea Nitrogen	12 mg/dL	6 - 20 mg/dL
	Creatine	1.2 mg/dL	0.4 - 1.3 mg/dL
	Calcium	10.5 mg/dL	8.5 - 10.2 mg/dL
Hemoglobin A1C	Hemoglobin A1C	13.7%	4 - 5.9 %

**Figure 1:**
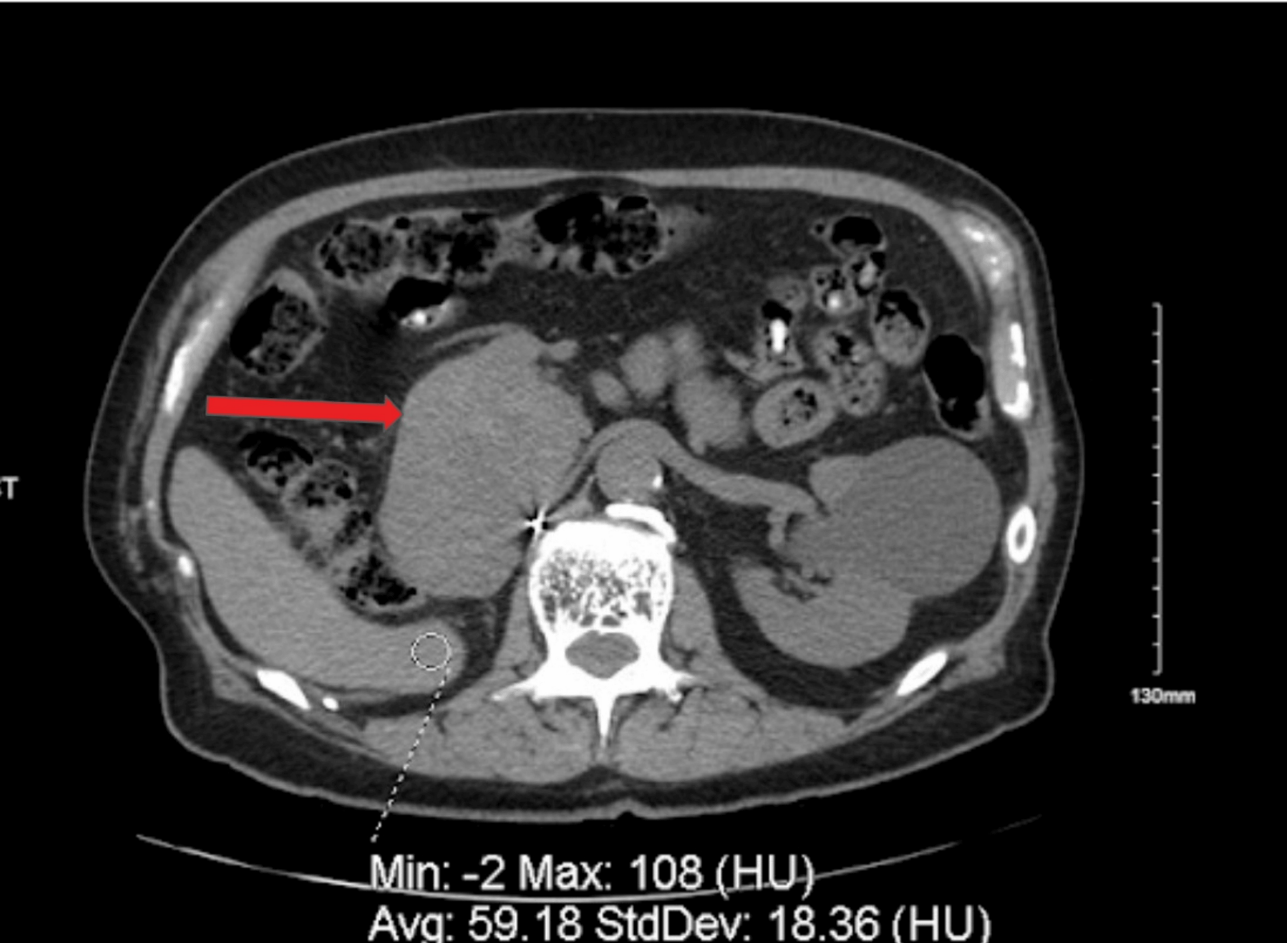
Axial abdominal computed tomography image The arrow indicates the pancreatic mass.

The initial assessment of the patient was new-onset type 2 diabetes mellitus with unintentional weight loss. The patient was started on metformin with close glucose monitoring. Given the rapid onset of hyperglycemia and significant weight loss, CT imaging (Figure 1) was obtained, which revealed a pancreatic mass, measuring 9.2 x 6.4cm with nodules in the right hepatic lobe and right lung, concerning for metastatic pancreatic cancer. 

**Figure 2:**
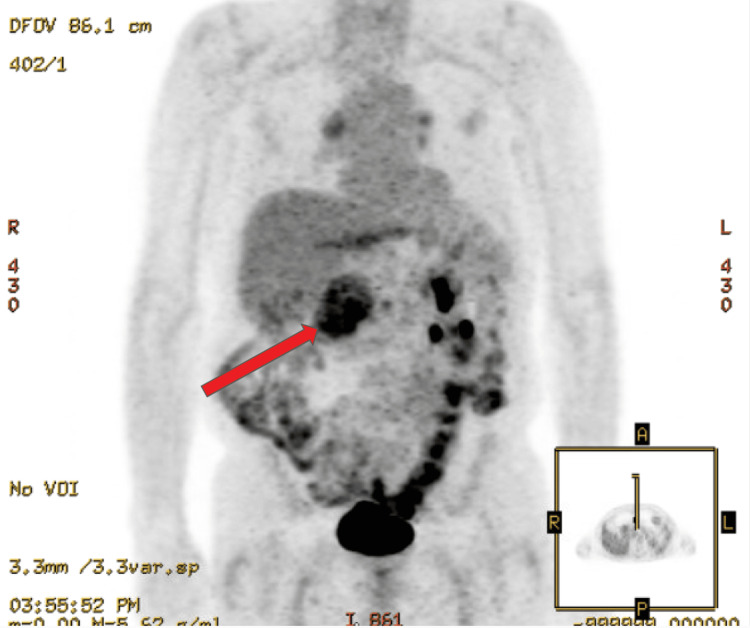
Coronal positron emission tomography image The arrow indicates the pancreatic mass.

**Figure 3:**
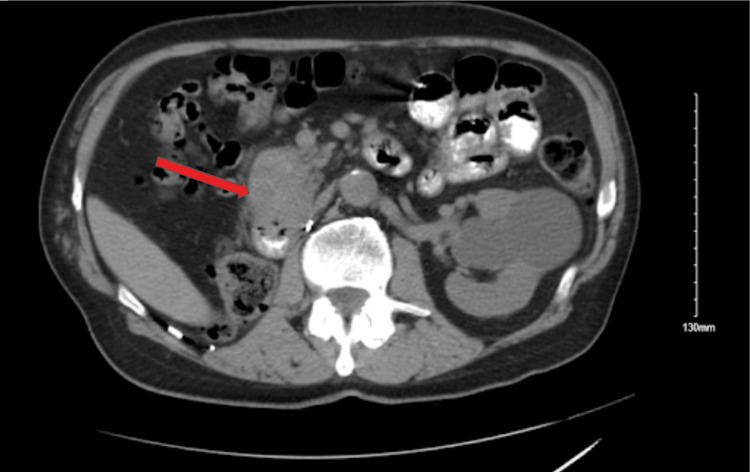
Axial abdominal computed tomography image The arrow indicates the pancreatic mass.

After initial imaging was completed, the patient returned to the clinic for a follow-up to discuss imaging results and options with regard to their diagnosis. He was started on metformin at the initial visit. At this time, the patient was referred to oncology for the imaging concerning metastatic pancreatic cancer and consented to the release of information regarding his case. A PET scan (Figure 2) and a biopsy of the head of the pancreas were obtained. The pathology report from the pancreatic biopsy revealed “metastatic clear cell renal cell carcinoma.” Following the pathology report, oncology started him on Inlyta 5mg P.O. BID and scheduled the patient for chemo port placement. Following placement of the port, the patient was started on Keytruda IV. As an assessment of the efficacy of the chemotherapy regimen, CT imaging (Figure 3) was obtained several months after starting chemotherapy which showed a decrease in the size of the mass in the head of the pancreas (from 9.2 x 6.4cm to 7.0 x 4.6cm), no evidence of the previously seen right lung mass, and no change in the size of the mass in the right hepatic lobe. The mass in the right hepatic lobe was identified as a probable hepatic cyst based upon “decreased attenuation.” Due to the evidence of chemotherapy reducing metastases, excision has been scheduled for the pancreatic mass.

## Discussion

Late recurrence of ccRCC after five years is uncommon, and recurrence occurring decades later is even rarer. While the majority of recurrences happen within the first 5 to 10 years after nephrectomy, cases of recurrence more than 30 years after initial surgery have been described in the literature [5]. Tapper et al. reported a case of recurrence 45 years after nephrectomy [5]. This finding underscores that extremely delayed relapse can occur, despite its rarity [3,4]. Given this rarity, investigation into other predisposing factors for the recurrence of ccRCC is warranted. 

Several factors may contribute to an increased likelihood of late recurrence in ccRCC. Evidence suggests that prolonged inflammatory states may increase the risk of developing renal malignancy and may contribute to the recurrence of ccRCC [9]. Yoshimura et al. surmised that inflammatory states, like those of rheumatoid arthritis, may contribute to recurrence and metastases even when immunotherapy is being used to control immune response [9]. In addition, several established risk factors for ccRCC overlap with conditions that accelerate chronic kidney injury. For example, diabetes and hypertension are associated with increased renal stress, progressive nephron loss, and renal remodeling, which may contribute to carcinogenesis and increase the risk of ccRCC [10]. Some studies also suggest shared genetic and metabolic pathways between type 2 diabetes and ccRCC [10]. Yang et al. suggest LST1 as a biomarker for efferocytosis between type 2 diabetes and ccRCC [11]. Given the multifaceted connection between type two diabetes and ccRCC, it is apparent that this relation is a part of this case presentation. 

The clinical presentation in this patient, new-onset diabetes leading to the diagnosis of recurrent ccRCC, is unusual. More commonly, diabetes is considered a risk factor that predisposes patients to the development of ccRCC [6-9]. However, pancreatic involvement from metastatic disease represents a plausible explanation for new-onset hyperglycemia in select cases. Although ccRCC is known to metastasize to endocrine organs such as the pancreas, pancreatic metastasis is far less common than spread to sites such as the lungs, bone, liver, and brain [1]. Pancreatic metastases account for only a small proportion of metastatic sites in ccRCC, occurring in approximately 2.8% of cases [4]. This pattern of metastatic spread provides a compelling explanation for this patient’s abrupt-onset diabetes and highlights pancreatic involvement as a clinically important but easily overlooked manifestation of recurrent ccRCC. 

Although rare, pancreatic metastasis of RCC has been reported in association with new-onset diabetes [12]. Jelleli et al. proposed that pancreatic metastasis was the likely cause of new-onset diabetes in the patient described in their case [12]. While a definitive causal relationship cannot be established from a single case, the temporal association observed in this patient supports a plausible link between pancreatic involvement and the abrupt onset of hyperglycemia. To date, no well-defined mechanism has been clearly established in the literature to explain how RCC metastases to the pancreas can cause diabetes, and direct evidence linking metastatic RCC to altered islet hormone production remains limited. We therefore propose a hypothesized pancreatogenic mechanism, by analogy to pancreatic ductal adenocarcinoma-associated diabetes, tumor progression may lead to hyperactivation of TGF-β signaling, inducing selective β-cell apoptosis and a reduction in β-cell mass [13,14]. This β-cell loss could result in impaired insulin secretion and subsequent systemic hyperglycemia [13]. The proposed mechanism is intended to provide biologic plausibility rather than establish causation, and further evaluation of this proposed mechanism may help explain this patient’s atypical presentation and better define the relationship between pancreatic metastasis and new-onset diabetes.

## Conclusions

This case demonstrates a rare and exceptionally delayed recurrence of ccRCC more than three decades after nephrectomy. It highlights that ccRCC can recur after prolonged disease-free intervals and may present through non-classic pathways, including new-onset diabetes temporally associated with pancreatic involvement. Although a definitive causal relationship cannot be established from a single case, this presentation underscores the importance of maintaining long-term oncologic vigilance and considering prior malignancy in the differential diagnosis of unexplained metabolic abnormalities.
